# A therapeutic target for CKD: activin A facilitates TGFβ1 profibrotic signaling

**DOI:** 10.1186/s11658-023-00424-1

**Published:** 2023-01-30

**Authors:** Asfia Soomro, Mohammad Khajehei, Renzhong Li, Kian O’Neil, Dan Zhang, Bo Gao, Melissa MacDonald, Masao Kakoki, Joan C. Krepinsky

**Affiliations:** 1 Division of Nephrology, Department of Medicine, McMaster University, Hamilton, Canada; 2 Department of Pathology and Laboratory Medicine, University of North Carolina, Chapel Hill, NC USA; 3 St. Joseph’s Hospital, 50 Charlton Ave East, Rm T3311, Hamilton, ON L8N 4A6 Canada

**Keywords:** Activin A, TGFβ1, Kidney fibrosis, Extracellular matrix

## Abstract

**Background:**

TGFβ1 is a major profibrotic mediator in chronic kidney disease (CKD). Its direct inhibition, however, is limited by adverse effects. Inhibition of activins, also members of the TGFβ superfamily, blocks TGFβ1 profibrotic effects, but the mechanism underlying this and the specific activin(s) involved are unknown.

**Methods:**

Cells were treated with TGFβ1 or activins A/B. Activins were inhibited generally with follistatin, or specifically with neutralizing antibodies or type I receptor downregulation. Cytokine levels, signaling and profibrotic responses were assessed with ELISA, immunofluorescence, immunoblotting and promoter luciferase reporters. Wild-type or TGFβ1-overexpressing mice with unilateral ureteral obstruction (UUO) were treated with an activin A neutralizing antibody.

**Results:**

In primary mesangial cells, TGFβ1 induces secretion primarily of activin A, which enables longer-term profibrotic effects by enhancing Smad3 phosphorylation and transcriptional activity. This results from lack of cell refractoriness to activin A, unlike that for TGFβ1, and promotion of TGFβ type II receptor expression. Activin A also supports transcription through regulating non-canonical MRTF-A activation. TGFβ1 additionally induces secretion of activin A, but not B, from tubular cells, and activin A neutralization prevents the TGFβ1 profibrotic response in renal fibroblasts. Fibrosis induced by UUO is inhibited by activin A neutralization in wild-type mice. Worsened fibrosis in TGFβ1-overexpressing mice is associated with increased renal activin A expression and is inhibited to wild-type levels with activin A neutralization.

**Conclusions:**

Activin A facilitates TGFβ1 profibrotic effects through regulation of both canonical (Smad3) and non-canonical (MRTF-A) signaling, suggesting it may be a novel therapeutic target for preventing fibrosis in CKD.

**Supplementary Information:**

The online version contains supplementary material available at 10.1186/s11658-023-00424-1.

## Introduction

The burden of chronic kidney disease (CKD) is large and growing, affecting 11–15% of the adult population [1]. CKD not only increases risk of kidney failure, but at all stages is also a major contributor to cardiovascular disease risk [2]. Multifactorial interventions including treatment of hypertension, inhibition of the renin-angiotensin system and SGLT2 inhibitors in patients with proteinuria only delay disease progression [3, 4]. The identification of new therapeutic agents to slow CKD progression is thus an important clinical challenge.

Regardless of etiology, CKD is characterized by fibrosis in all kidney compartments, with the secreted cytokine TGFβ1 well established as its central profibrotic mediator [5]. Direct inhibition of TGFβ1 with neutralizing antibodies, however, is limited by adverse effects [6], likely due to its important role in homeostasis. Recently, activins have emerged as potential important mediators of TGFβ1 profibrotic effects and may thus represent an alternative antifibrotic target.

Activins are multifunctional secreted cytokines belonging to the TGFβ superfamily which, like TGFβ1, also promote fibrosis [7, 8]. They are formed as homo- or heterodimers of inhibin β subunits A, B, C or E. Activin A (actA), a homodimer of the A subunit, is the most widely studied [9]. Both actA and B are expressed in the injured kidney [10, 11], with actC and E mainly expressed in the liver [12, 13]. ActA/B signaling is analogous to that of TGFβ, occurring through heteromeric complexes of type I and II transmembrane serine/threonine kinase receptors [9]. These activate Smad2/3 proteins through phosphorylation, leading to their nuclear translocation to regulate transcription of target genes. TGFβ1 and activins use different type I and II receptors. For activins, these are ActRIIA or ActRIIB and the type I receptor ALK4 for actA and ALK7 for actB [9]. Various Smad-independent pathways also contribute importantly to TGFβ1 and activin signaling [5].

Follistatin is a potent inhibitor of activins which does not neutralize TGFβ1 [14]. Intriguingly, however, TGFβ1-induced matrix production is inhibited by follistatin in multiple cell types including glomerular mesangial cells (MC) and renal fibroblasts [15–18]. Furthermore, actA and TGFβ1 augment each other’s expressions [15–17, 19], suggesting that actA may be a major mediator of TGFβ1 profibrotic effects. However, follistatin also inhibits other TGFβ superfamily ligands, albeit with significantly lower potency [7, 9, 14], and studies to date have not directly examined the specific contribution of particular activins to TGFβ1 profibrotic effects. Furthermore, how activins would enable this, given that activins and TGFβ1 use the same intracellular protein mediator Smad3, is as yet unknown. Here we thus aim to elucidate the relative importance of actA and B in TGFβ1-induced profibrotic effects and the mechanism by which this might occur. We further investigate the efficacy of actA neutralization in attenuating kidney fibrosis in mice overexpressing TGFβ1.

## Methods

### Cells

Primary glomerular mesangial cells (MC) from B6129SF1/J male mice were cultured in DMEM with 20% FBS and streptomycin/penicillin. Rat primary renal fibroblasts (Cell Biologics) and human kidney 2 (HK2) proximal tubular cells were cultured in 1:1 DMEM/F12 with 10% FBS and streptomycin/penicillin. Primary cells were used at passages 10–17. Cells were serum deprived at 80–90% confluence in 1% BSA for 24 h prior to treatment with: 0.5 ng/ml TGFβ1 (the lowest dose identified in our studies to produce consistent responses), 0.5 ng/ml TGFβ3, 2 or 20 ng/ml actA, 2–5 ng/ml actB, 1 µg/ml SIS3 (Cayman), 500 ng/ml follistatin (the lowest dose showing consistent inhibition in our studies), 3.5 µg/ml anti-actA antibody, 2.5 µg/ml anti-actB or control IgG antibody (all R&D Systems) for the times indicated.

### Protein analysis

Protein was extracted and prepared for immunoblotting as per standard protocol. For nuclear protein, cells were lysed in hypotonic buffer. After centrifugation, pelleted nuclei were sonicated in hypotonic buffer with 0.4 M NaCl and 10% glycerol, centrifuged and supernatant with nuclear protein used.

To isolate cell surface proteins, MC were incubated with 1 mg/ml EZ-link Sulfo-Biotin (Pierce) for 30 min, then washed with 0.1 M glycine in PBS, lysed, clarified, and equal quantities of protein incubated overnight in a 50% Neutravidin slurry (Thermo Fisher) to capture biotin-tagged proteins. Beads were washed, boiled for 10 min in 2×PSB and cell surface proteins assessed by immunoblotting.

For immunoblotting, equal amounts of protein were electrophoresed and transferred onto nitrocellulose membranes. Primary antibodies were: fibronectin (BD Transduction; 610078), αSMA (Pierce; MA1-06110), CTGF (Sigma; AMAB91366), Smad3 (Abcam; ab40854), pSmad3 (Novus; NBP1-77836), pSmad2 (Cell Signaling; 3108), Smad2/3 (Cell Signaling; 8685) MRTF-A (Abcam; ab49311), GAPDH (Millipore; CB1001), TRI (Abcam; ab31013), TRII (Abcam; ab78419), ALK4 (Abcam, ab109300) PDGFR (Cedarlane; 1469-1), tubulin (Invitrogen; 32-2700) and lamin B (Santa Cruz; ac-6217). ImageJ was used to quantify band intensity. The following horseradish peroxidase conjugated secondary antibodies were used: goat (BioRad; 1721034), mouse (BioRad; 170-6516) and rabbit (BioRad; 170-6515).

Media actA and B or serum actA were measured using ELISA (R&D).

### Collagen gel contraction

Hydrated collagen gels were prepared by mixing 5 × 10^5^ rat renal fibroblasts with 3.5 mg/ml of rat tail type I collagen in DMEM-F12 (Advanced BioMatrix). The mixture was poured into 30 mm-diameter moulds and incubated for an hour at 37 °C in a humidified atmosphere to allow the gel to polymerize before transferring to 35-mm diameter tissue culture plates with media. Fibroblast-populated gels were then treated with TGFβ1 with or without follistatin or anti-ActA antibody for 72 h and then photographed. The radius of each gel was measured using Digimizer software, with measurements normalized to the diameter of the well. The radius of each gel was normalized to the initial known radius of the mould (30 mm).

### Transfection

Approximately 7 × 10^5^ MCs were seeded to achieve 60–70% confluence prior to transfection with 0.5 μg of either CAGA_12_ luciferase (12 repeats of the Smad3-resposive element) or αSMA luciferase reporter (pGal3-α-SMAp-luc) gifted by Dr. A. Kapus (University of Toronto, Canada) and 0.05 μg pCMV β-galactosidase (Clonetech) using Effectene (Qiagen). After harvest, luciferase and β-galactosidase activities were measured using the respective kits (both Promega).

1 × 10^4^ MCs were seeded onto an 8-well chamber slide to transiently express eGFP-Smad3, gifted by Dr. X. Fang, The Chinese Academy of Sciences [20], using electroporation (250 V, 30 ms) with the ECM830 square wave electroporator.

For siRNA transfections, 4 × 10^5^ MCs were seeded on a 6-well plate to attain 30–40% confluence. ALK4 siRNA (50 nM; Thermo Fisher) knockdown was achieved using RNAiMAX (Thermo Fisher).

### Immunofluorescence

MC grown on chamber slides were transfected with eGFP-Smad3. After treatment, cells were washed, fixed with 4% paraformaldehyde and stained with DAPI before coverslips were placed. Slides were imaged at 40× magnification using an Olympus IX81 fluorescence microscope with Metamorph. Signal intensity in 30 random nuclei was quantified, averaged for 3 independent experiments.

### PCR

RNA was extracted using TRIzol (Invitrogen) and 1 µg was reverse transcribed to cDNA using qScript cDNA SuperMix Reagent (Quanta Biosciences) for quantitative real-time PCR using Power SYBR Green PCR Master Mix (Thermo Fisher) on the Applied Biosystems ViiA 7 Real-Time PCR system. Primers were: TRII F5′-GGTCTATGACGAGCGACGGG-3′, R5′-GCTTCCATTTCCACATCCGAC-3′; TGFβ1 F5′-AAACGGAAGCGCATCGAA-3′, R5′-GGGACRGGCGAGCCTTAGTT-3′, fibronectin F5′-GATGGAATCCGGGAGCTTTT-3′, R5′-TGCAAGGCAACCACACRGAC-3′; collagen Iα1 F5′-CTTCACCTACAGCACCCTTGTG-3′, R5′-GATGACTGTGCTTGCCCCAAGTT-3′; αSMA F5′-GACGCTGAAGTATCCGATAGAAC-3′; R5′-GGCCACACGAAGCTCGTTAT-3′; ALK4; F5′-CTGTTTGATTATCTGAACCG-3′; R5′-ACAACCTTTCGCATCTCCTC-3′; ACRIIA; F5′-GTTGAACCTTGCTATGGTGATAA-3′; R5′-AATCAGTCCTGTCATAGCAGTTG-3′; ACRIIB F5′-CACAAGCCTTCTATTGCCCACAG-3′; R5′-ATFTACCGTCTGGTGCCAAC-3′. Gene expression was calculated using the ∆∆C_T_ method with 18S (F5′-GCCGCTAGAGGTGAAATTCTTG-3′, R5′-CATTCTTGGCAAATGCTTTCG-3′) used as an internal control.

### Migration and proliferation assays

To assess migration, 1.5 × 10^5^ MCs were seeded per well on a 6-well plate. After 24 h of serum starvation, a scratch was made across the diameter of the well with a 1 ml pipette tip immediately before treatment. The area of the scratch was measured after 24 h under transmitted light using ImageJ. To assess proliferation, cells were seeded at 1.5 × 10^5^ MCs per well on a 6-well plate and serum deprived the next day for 24 h. They were then treated for 24 h after which cells were trypsinized and counted.

### Animal studies

These were carried out in accordance with the principles of laboratory animal care and McMaster University and Canadian Council on Animal Care guidelines. C57BL/6 mice with hypermorphic alleles for TGFβ1 (resulting in ~ 300% normal expression) and their genotyping were described previously [21]. Wild-type (WT) mice were from Charles River. Male mice aged 8 weeks underwent unilateral ureteral obstruction, achieved by left ureteral ligation close to the renal pelvis. Sham mice were anesthetized and the kidney manipulated without ligation. Mice were treated with vehicle, 3 μg anti-actA antibody (MAB3381, R&D) or IgG1 (AF007, R&D) intraperitoneally daily until harvest at day 10 when weight, serum and left kidney were obtained. This dose of antibody was extrapolated from a previous study investigating endometriosis-induced fibrosis in mice. Here, the antibody inhibited fibrosis after 4 weeks of treatment, without observable toxicity [22].

### Kidney processing

Protein was extracted from liquid nitrogen-stored kidney cores using T-PER™ reagent (Thermo Fisher) with protease/phosphatase inhibitor tablets (Roche). Tissue was homogenized using 0.5 g Lysing Matrix D beads and the Bead Ruptor Elite homogenizer (5 m/s, 30 s), centrifuged and supernatant used.

For immunohistochemistry, formalin-fixed, paraffin-embedded kidneys were sectioned at 4 μm and stained with picrosirius red (Polysciences), Masson trichrome (Sigma), αSMA (Pierce) and pSmad3 (Novus). Imagestaken at 20× using the BX41 Olympus microscope, or for PSR using the Olympus IX81 fluorescence microscope, were analysed using ImageJ. All were quantified by measuring the percentage of positive area.

For Masson trichrome, areas of collagen fibre stained with blue dye were thresholded for colour, saturation, and brightness. PSR stained sections were inverted to monochrome pictures before measuring positive areas. The positive area was divided by the total area for each field, and the average value was calculated for each mouse section to yield one value per section.

### Statistical analysis

Values are presented as mean ± SEM. Statistical difference among multiple groups was determined using a one-way ANOVA with a Tukey’s post hoc test. Unpaired, two-tailed Student *t* tests were used for single comparisons. P values < 0.05 were considered significant using GraphPad Prism 7 for calculations.

## Results

### Activin inhibition attenuates TGFβ1-induced fibrotic responses and Smad3 activation

**Fig. 1:**
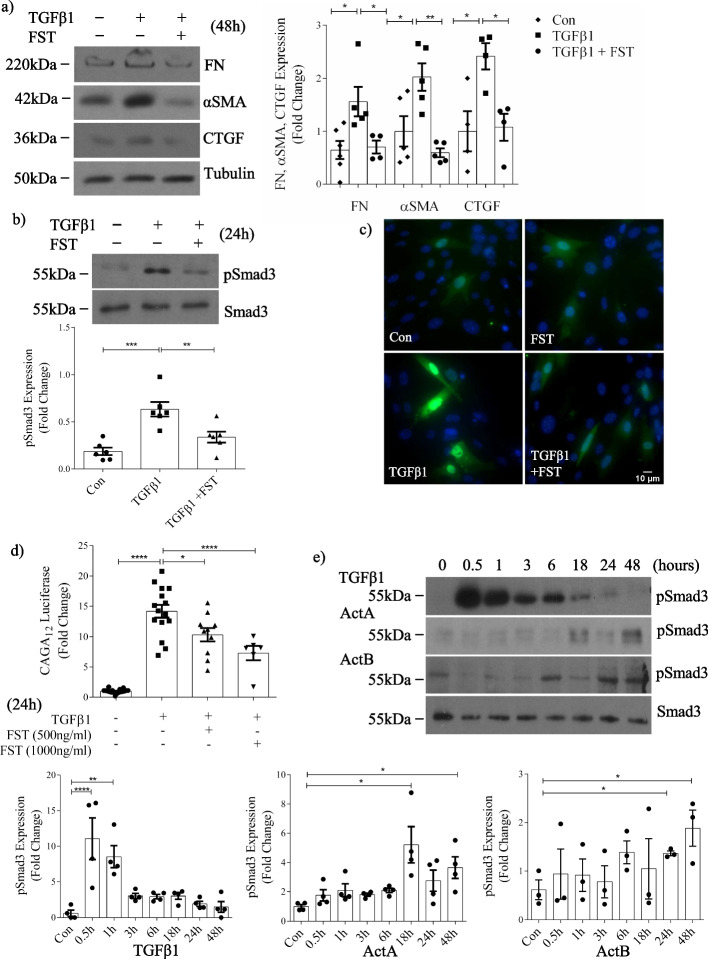
Activin inhibition attenuates TGFβ1-induced fibrotic responses and Smad3 activation in MC. Activin inhibition with follistatin (FST) decreases TGFβ1-induced: **a** FN, αSMA and CTGF upregulation at 48 h (n = 5), **b** Smad3 phosphorylation (pSmad3) at 24 h (n = 5), **c** Smad3 nuclear translocation as assessed using eGFP-Smad3 (n = 3; 25–30 cells quantified per treatment group) at 24 h, and **d** Smad3 transcriptional activity at 24 h (n = 8). **e** Time course experiments show increases in pSmad3 occur earlier (30–60 min) with TGFβ1 (n = 4) compared with actA (n = 4) or actB (n = 3) (18–48 h). *, **, ***, ****P < 0.05, 0.01, 0.001, 0.0001; one-way ANOVA with Tukey’s multiple comparisons post hoc test

We first confirmed that activin inhibition with follistatin could prevent TGFβ1 profibrotic responses in MC. As shown in Fig. 1a, TGFβ1-induced expression of the matrix protein fibronectin and profibrotic cytokine connective tissue growth factor (CTGF) were inhibited by follistatin. The induction of α-smooth muscle actin (αSMA), characteristic of a profibrotic MC phenotype, was also inhibited. Follistatin does not directly neutralize TGFβ1. We confirmed this in MC for early (30-min) TGFβ1-induced Smad3 activation as assessed by its phosphorylation (Additional file 1: Fig. S1). Intriguingly, however, follistatin attenuated later (24-h) Smad3 activation by TGFβ1 (Fig. 1b). Correspondingly, nuclear translocation of green fluorescent protein (GFP)-tagged Smad3 in response to TGFβ1 was also attenuated by follistatin (Fig. 1c), as was Smad3 transcriptional activity which was assessed using the Smad3-responsive CAGA_12_ luciferase reporter (Fig. 1d). Since follistatin most potently inhibits activins A and B, we next determined the time courses of Smad3 activation in response to these activins and TGFβ1. Figure 1e shows that TGFβ1 leads to early Smad3 activation which is maximal at 30 min. Smad3 activation in response to both activins occurs much later, at 18–48 h when the TGFβ1-induced response is approaching baseline. It should be noted that two bands are seen in some blots for pSmad3, dependent on film exposure time. Our previous studies using Smad3 knockout MC have verified that it is the bottom band that represents pSmad3.

Finally, we determined whether follistatin could also inhibit renal fibroblast responses to TGFβ1. As shown in Additional file 1: Fig. S2, TGFβ1-induced Smad3 activation and upregulation of αSMA and matrix proteins were also inhibited by follistatin in these cells, showing a more generalized requirement for activins in TGFβ1-induced longer-term Smad3 signaling and profibrotic responses.

### Specific actA inhibition attenuates TGFβ1-induced Smad3 activation and profibrotic responses

**Fig. 2:**
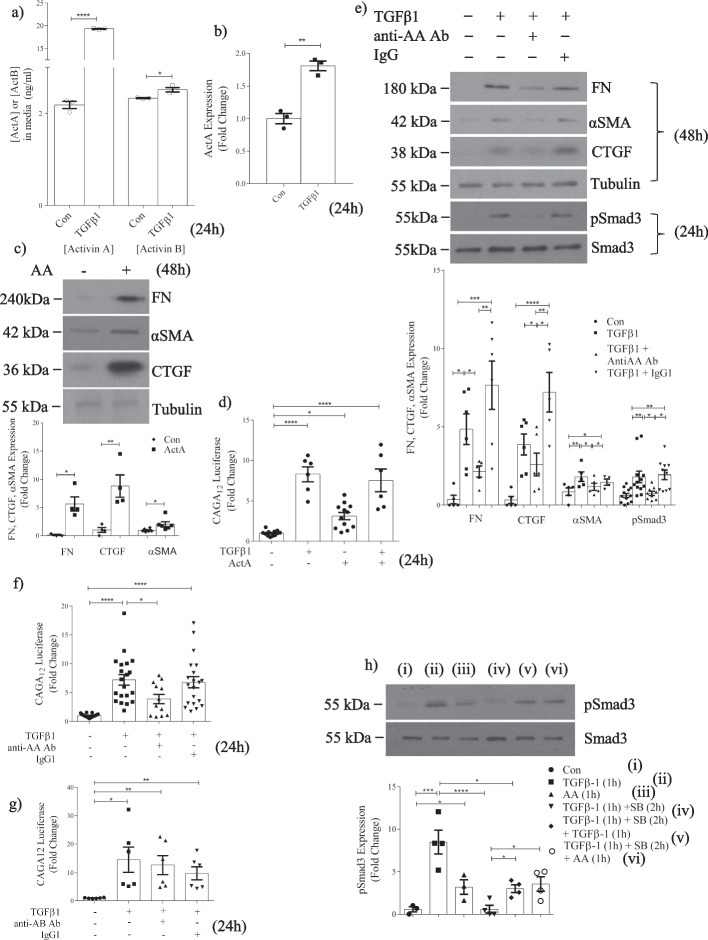
Specific activin A inhibition attenuates TGFβ1-induced Smad3 activation and profibrotic responses in MC. **a** ELISA demonstrates that TGFβ1 (24 h) increases actA and actB secretion (n = 3) to 19.5 ng/ml and 2.5 ng/ml, representing an 8.9- and 1.06-fold induction respectively. **b** TGFβ1 increases actA in whole cell lysate by 1.8-fold (n = 3). **c** ActA (20 ng/ml) upregulates FN (n = 3–4), CTGF (n = 4) and αSMA (n = 6) at 48 h. **d** TGFβ1 and actA both increase Smad3 transcriptional activity; no synergistic effect is seen (n = 6–12). **e** An actA neutralizing antibody attenuates TGFβ1-induced FN (n = 5–6), αSMA (n = 5), CTGF (n = 5–6), and Smad3 activation (n = 10–12). **f** ActA neutralization decreases TGFβ1-induced Smad3 transcriptional activity at 24 h (n = 9–15), but this is not decreased by actB neutralization (n = 6) (**g**). **h** MC were stimulated with TGFβ1 or actA for 1 h, then treated with their type I receptor inhibitor SB431542 (50 µM). Restimulation with the same ligand shows that cells become refractory to TGFβ1, but not actA (n = 4). *, **, ***, ****P < 0.05, 0.01, 0.001, 0.0001; one-way ANOVA with Tukey’s multiple comparisons post hoc test

Our data raise the possibility that TGFβ1 increases activin secretion to maintain longer-term Smad3 activation. Indeed, Fig. 2a shows that TGFβ1 increases actA and actB secretion into the medium after 24 h by 8.9- and 1.06-fold (to 19.5 and 2.5 ng/ml) respectively. Although both were statistically significant increases, our further studies focused on the role of actA given its far greater induction. We used actA at 20 ng/ml for subsequent studies as this approximated the concentration seen in the media in response to TGFβ1. Figure 2b shows that cellular actA, assessed by ELISA, was also increased by TGFβ1 by 1.8-fold at 24 h. Figure 2c shows that 20 ng/ml actA induced fibronectin, αSMA and CTGF upregulation at 48 h, confirming that secreted levels of actA exert profibrotic effects.

Both activins and TGFβ1 signal through the same Smad proteins. We thus tested whether their effects were additive for Smad3 activity. Figure 2d shows that while each increased Smad3 transcriptional activity, their combination did not further enhance this. Next, we used a neutralizing antibody to determine the specific role of actA in TGFβ1 profibrotic responses. We first confirmed that it specifically inhibited actA, but not actB signaling (Additional file 1: Fig. S3). Figure 2e shows this antibody reduced the upregulation of fibronectin, αSMA and CTGF after 48 h of TGFβ1 while nonspecific IgG had no effect. No effects on basal levels of protein expression were seen with either follistatin or actA neutralization (not shown). Inhibition was also seen at the transcriptional level. Additional file 1: Fig. S4 shows attenuation of the TGFβ1-induced increase in fibronectin, collagen Iα1 and αSMA transcripts by actA neutralization. Finally, Smad3 phosphorylation and transcriptional activity (Fig. 2e, f) were additionally significantly attenuated, showing that inhibition of later Smad3 activation by follistatin in response to TGFβ1 is largely recapitulated by specific actA inhibition. We next tested the effect of an actB neutralizing antibody. This did not alter TGFβ1-induced Smad3 transcriptional activity (Fig. 2g), in keeping with its minimal increase by TGFβ1.

Finally, we determined whether the requirement for actA in TGFβ1 profibrotic effects is also seen in renal fibroblasts. Additional file 1: Fig. S2 shows that actA neutralization similarly attenuates TGFβ1-induced Smad3 activation and upregulation of fibrotic markers in these cells. To assess the functional importance of this finding, we tested the effects of actA inhibition on TGFβ1-induced fibroblast behaviour important in promoting remodeling and fibrosis. Additional file 1: Fig. S5 shows that both follistatin and actA neutralization attenuated TGFβ1-induced proliferation, migration, and collagen gel contraction. These observations suggest that actA supports TGFβ1 in promoting fibroblast remodeling behaviour.

The rapid internalization of receptors after acute TGFβ1 stimulation renders cells refractory to further stimulation [23]. We postulated that actA may maintain Smad3 activation during this time of cell refractoriness to TGFβ1. We thus investigated MC responsiveness to TGFβ1 or actA stimulation and restimulation. Cells were treated with ligands for 1 h, then washed and treated with the type I receptor kinase inhibitor SB431542 to prevent further ligand signaling. They were then restimulated with ligand for 1 h. As seen in Fig. 2h, Smad3 activation was significantly reduced after TGFβ1 restimulation, but was maintained after actA restimulation. These data show that cells remain responsive to actA restimulation and suggest that TGFβ1 may rely on actA over time to assist in sustaining Smad3 signaling.

### Activin A facilitates type II receptor upregulation by TGFβ1

**Fig. 3:**
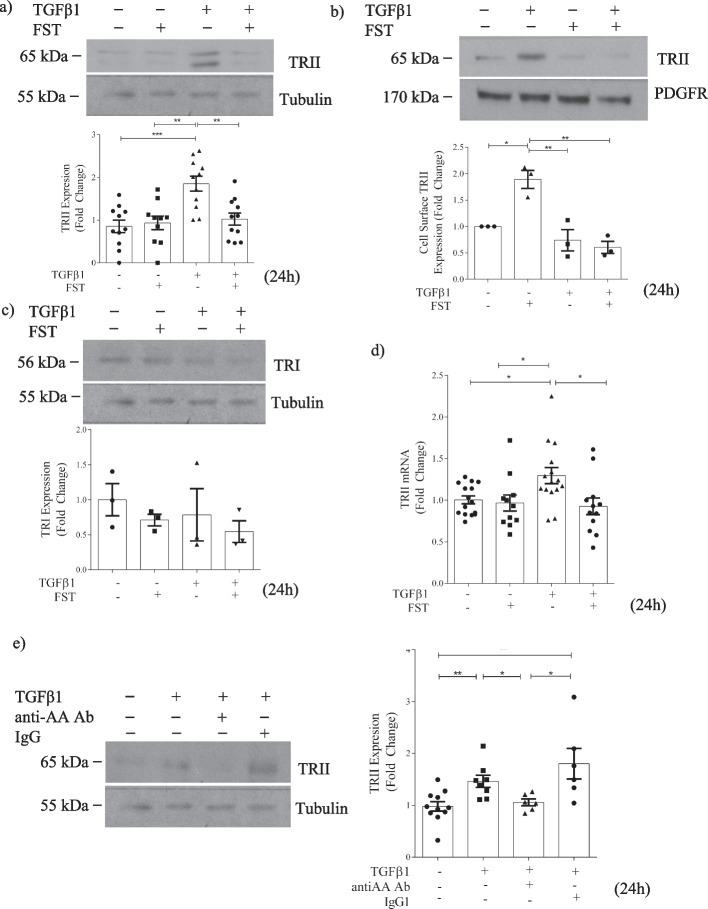
Activins facilitate TGFβ1 receptor type II upregulation in response to TGFβ1 in MC. **a** Follistatin (FST) inhibits the TGFβ1-induced increase in TRII expression at 24 h in whole cell lysate (n = 10–11). **b** The TGFβ1-induced increase in cell surface TRII was diminished by follistatin at 24 h (n = 3). **c** No change in response to either TGFβ1 or follistatin were seen with TRI (n = 6). **d** The TGFβ1-induced increase in TRII transcript was also diminished by follistatin at 24 h (n = 13–15). **e** TGFβ1 induction of TRII expression is reduced with an actA neutralizing antibody (n = 6). *, **, ***, ****P < 0.05, 0.01, 0.001, 0.0001; one-way ANOVA with Tukey’s multiple comparisons post hoc test

We next asked whether actA might regulate TGFβ1 receptor levels. When cotreated for 24 h, follistatin significantly attenuated TGFβ1-induced increase in TRII in both whole cell lysate (Fig. 3a) and at the cell surface (Fig. 3b), with neither treatment altering TRI (Fig. 3c). This appeared to be a transcriptional effect, since follistatin coincubation prevented the increase in TRII transcript by TGFβ1 (Fig. 3d). Specific inhibition of actA using a neutralizing antibody similarly prevented the TGFβ1-induced increase in whole cell lysate TRII compared to nonspecific IgG (Fig. 3e). These results suggest that inhibition of actA attenuates TGFβ1 signaling by reducing TRII available to bind ligand.

**Fig. 4:**
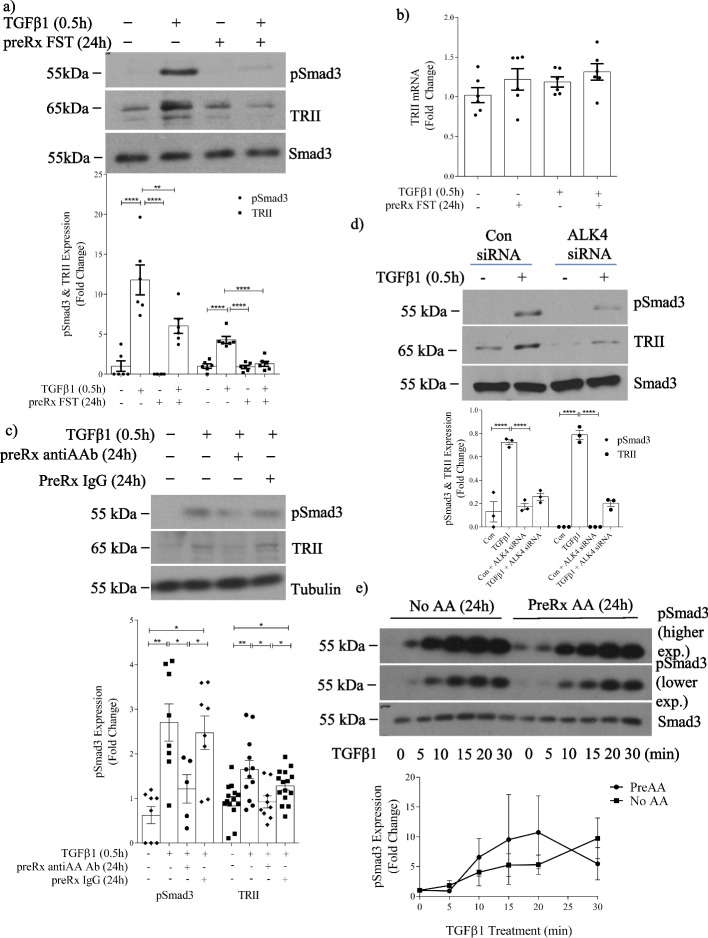
Activin A facilitates TGFβ1-induced type II receptor expression without altering TGFβ1 receptor sensitivity in MC. **a** TGFβ1 for 30 min increased TRII expression and Smad3 activation in whole cell lysate, and both were prevented by 24 h pretreatment with follistatin (n = 6). **b** There was no change in TRII transcript after 24 h of follistatin with or without TGFβ1 for 30 min. **c** Pretreatment with an actA neutralizing antibody for 24 h inhibited acute (30 min) TGFβ1-induced Smad3 activation and TRII upregulation (n = 6–7). These were similarly inhibited by downregulation of the actA type I receptor ALK4 with siRNA (**d**) (n = 3). **e** Pretreatment with actA (20 ng/ml) for 24 h did not affect acute TGFβ1-induced Smad3 activation (n = 6). *, **, ****P < 0.05, 0.01, 0.0001; one-way ANOVA with Tukey’s multiple comparisons post hoc test

We next wished to determine whether initial 24 h activin inhibition would alter acute TGFβ1 signaling. We thus incubated cells with follistatin for 24 h followed by TGFβ1 for 30 min. Interestingly, contrary to the lack of inhibition of TGFβ1-induced Smad3 activation with short-term (30 min) activin inhibition (Additional file 1: Fig. S1), 24 h follistatin preincubation completely abrogated Smad3 activation by TGFβ1 (Fig. 4a). Furthermore, TRII levels were increased by TGFβ1 even after only 30 min and this was also prevented by follistatin preincubation (Fig. 4a). Two bands are seen for TRII. The higher band represents the mature, more predominant and more stable glycosylated TRII species. The lower band represents the precursor form [24]. TRII transcript was not increased at this time (Fig. 4b), indicating a posttranscriptional mechanism for its upregulation. These data suggest that basal activin levels are important for TGFβ1 responsiveness. Both the increase in TRII and Smad3 activation could also be blocked by either specific neutralization of actA (Fig. 4c) or siRNA downregulation of the actA type I receptor ALK4 (Fig. 4d), supporting a specific role of actA. Finally, to determine whether actA could alter sensitivity to TGFβ1, we assessed Smad3 activation after actA pretreatment for 24 h followed by TGFβ1 for 5 to 30 min. No difference was seen, suggesting that increasing actA beyond basal levels does not alter acute TGFβ1 responses (Fig. 4e). This is also in keeping with the lack of inhibition of acute TGFβ1-induced Smad3 activation by follistatin (Additional file 1: Fig. S1), showing that initial TGFβ1 activation of Smad3 is activin-independent. With longer treatment time, cells become refractory to TGFβ1 signaling while actA responsiveness is maintained.

### Activin A regulation of MRTF-A contributes to αSMA induction by TGFβ1

**Fig. 5:**
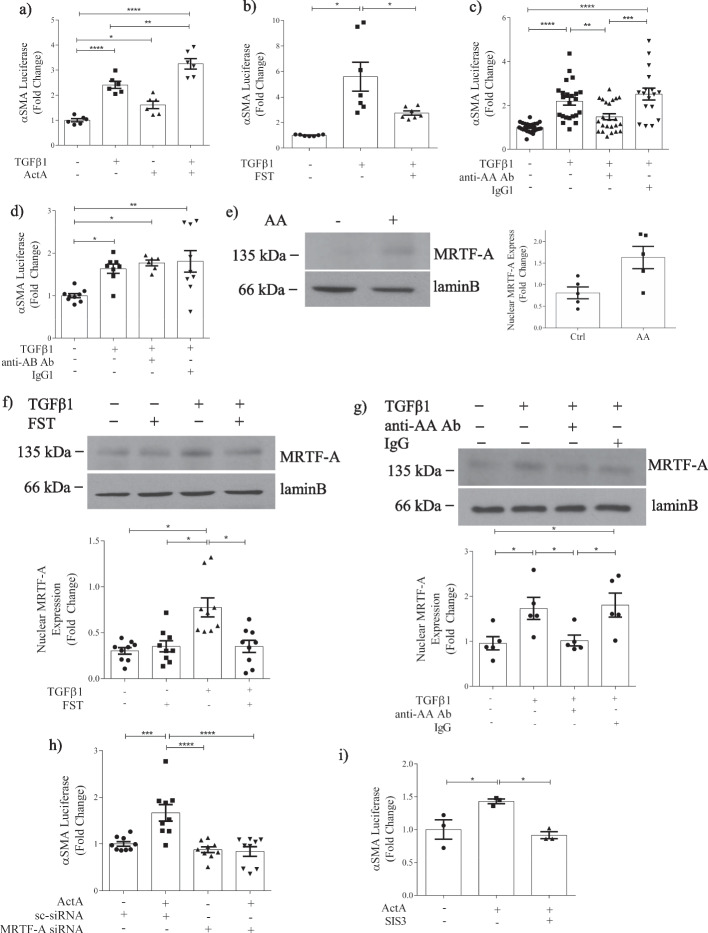
Activin A regulation of MRTF-A contributes to αSMA induction by TGFβ1 in MC. **a** ActA (20 ng/ml) and TGFβ1 show an additive effect on αSMA promoter transcriptional activation at 24 h (n = 6). Activin inhibition with **b** follistatin (n = 7) and **c** an actA neutralizing antibody decrease TGFβ1-induced αSMA promoter transcriptional activity (n = 14–23). **d** actB neutralization does not decrease TGFβ1-induced αSMA promoter transcriptional activity (n = 6–9). **e** actA increases MRTF-A nuclear localization at 18 h (n = 5). TGFβ1-induced nuclear MRTF-A translocation is attenuated with **f** follistatin (n = 9) and **g** actA neutralization (n = 5). **h** ActA-induced αSMA promoter transcriptional activity is inhibited by MRTF-A siRNA (n = 9) and **i** the Smad3 inhibitor SIS3 (n = 3). *, **, ***, ****P < 0.05, 0.01, 0.001, 0.0001; one-way ANOVA with Tukey’s multiple comparisons post hoc test

Having established a role for actA in canonical TGFβ1 signaling, we next sought to determine whether it also regulated noncanonical responses. The induction of αSMA, a marker of activated, profibrotic MC, is a well characterized TGFβ1 effect requiring both Smad3 and non-Smad3 signaling. We first tested αSMA promoter activity. Figure 5a shows that actA itself increases promoter activity, although less effectively than TGFβ1. Unlike Smad3 activation, actA and TGFβ1 had an additive effect on αSMA promoter induction. Furthermore, greater inhibition of TGFβ1-induced αSMA promoter activation was seen with follistatin (Fig. 5b) compared to its effects on Smad3 transcriptional activity shown in Fig. 1d. These data suggest that actA induces additional non-canonical pathway activation. The specific role of actA was confirmed in Fig. 5c in which actA neutralization attenuated TGFβ1-induced αSMA promoter activity. ActB neutralization had no effect, confirming that actB does not contribute significantly to TGFβ1 profibrotic effects (Fig. 5d).

An interaction between canonical and noncanonical TGFβ1 signaling is well known, with cooperation between Smad3 and MRTF-A shown to regulate αSMA induction by TGFβ1 [25]. MRTF-A is a transcription factor that is retained in the cytoplasm by G-actin. Actin polymerization releases it to enter the nucleus and bind CArG box sequences in cooperation with serum response factor [26]. We thus determined whether actA could induce MRTF-A activation. Figure 5e shows that actA increased nuclear MRTF-A. Furthermore, the TGFβ1-induced nuclear translocation of MRTF-A was prevented by follistatin (Fig. 5f) and actA neutralization (Fig. 5g). These data show that actA regulates noncanonical signaling pathways. We next showed that actA itself is able to induce αSMA transcriptional activity (Fig. 5h) and this also depends on MRTF-A, given its inhibition by MRTF-A downregulation with siRNA. To determine whether Smad3 is also required for αSMA transcriptional activity by actA, Smad3 was inhibited with SIS3. This prevented actA-induced αSMA luciferase activation (Fig. 5i). Taken together, these data show that actA-induced MRTF-A activation, in conjunction with Smad3 regulation, contributes importantly to TGFβ1 transcriptional responses.

### Activin A neutralization inhibits renal fibrosis in TGFβ1-overexpressing mice

**Fig. 6:**
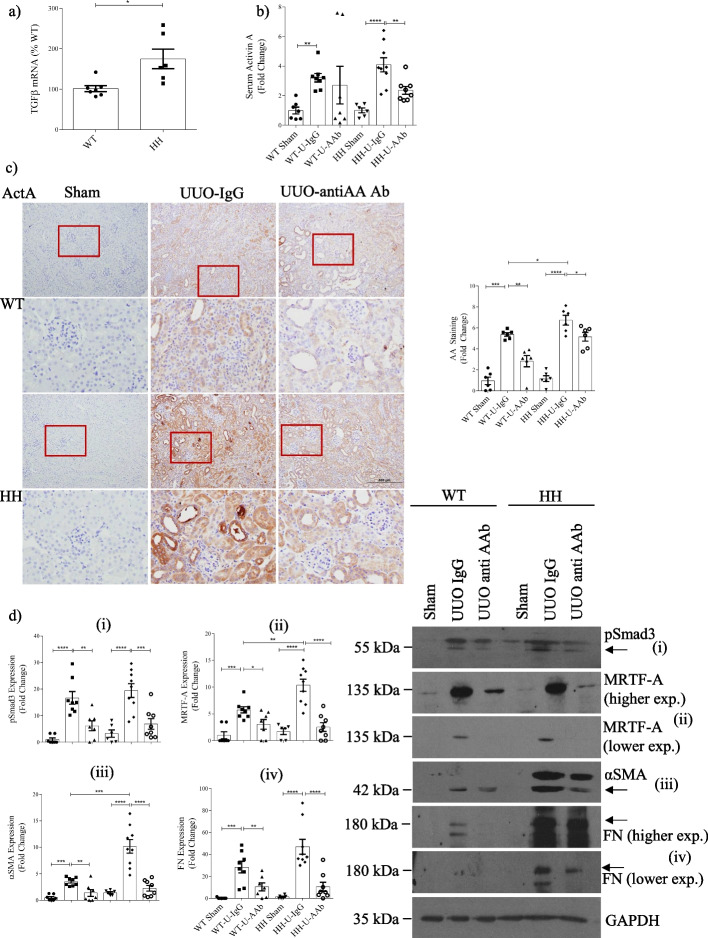
Activin A neutralization inhibits renal fibrosis in TGFβ1-overexpressing mice. **a** TGFβ1 transcript is increased in mice genetically engineered to overexpress TGFβ1 (HH) compared with wild-type mice (WT) (n = 6–7, *p ≤ 0.05). **b** Serum actA is elevated in wild-type and HH mice after UUO. This is decreased by treatment with a neutralizing actA antibody (anti-actA) in HH mice. **c** Renal actA is increased after UUO, with a greater induction in HH mice. Both are attenuated by actA neutralization. Boxed areas are shown at higher magnification immediately below. ActA increases are seen particularly in tubular epithelial cells. **d** Renal αSMA, fibronectin (FN), pSmad3 and MRTF-A are increased after UUO and this is augmented in HH kidneys. Expression of all is attenuated by actA neutralization in both WT and HH kidneys. (n = 6–9) *, **, ***, ****P < 0.05, 0.01, 0.001, 0.0001; one-way ANOVA with Tukey’s multiple comparisons post hoc test where there are > 2 groups; t-test for 2 groups

We next sought to determine the relevance of activin A to TGFβ1-induced fibrosis in vivo*.* For this, we used mice genetically manipulated to overexpress the TGFβ1 transcript at 300% of normal levels (denoted H/H), which have been previously described [27]. We confirmed increased TGFβ1 mRNA in H/H kidneys (Fig. 6a). These mice are on a C57BL/6 background which is relatively resistant to the development of renal fibrosis [28], with the exception of the unilateral ureteral obstruction (UUO) model [29]. We confirmed that TGFβ1 increased actA (but not B) transcript production and protein secretion in tubular cells in a dose-dependent manner (Additional file 1: Fig. S6), and that the TGFβ1-induced profibrotic response is also actA-dependent in renal fibroblasts (Additional file 1: Fig. S2), supporting the generalizability of MC findings to other renal cell types relevant to fibrosis. We thus used the UUO model to assess the efficacy of activin A neutralization on inhibiting the development of fibrosis.

UUO was induced in either WT or TGFβ1-overexpressing mice, with both groups treated with a neutralizing actA antibody (anti-actA) or control IgG for 10 days. We first assessed actA levels in both serum and kidneys. As seen in Fig. 6b, while UUO increased serum actA as previously shown by others [30], this was unaffected by TGFβ1 overexpression. However, the increased renal actA seen after UUO was augmented in H/H mice (Fig. 6c). No difference in renal actA was seen at baseline between genotypes. Treatment with anti-actA lowered both serum and renal actA levels.

**Fig. 7:**
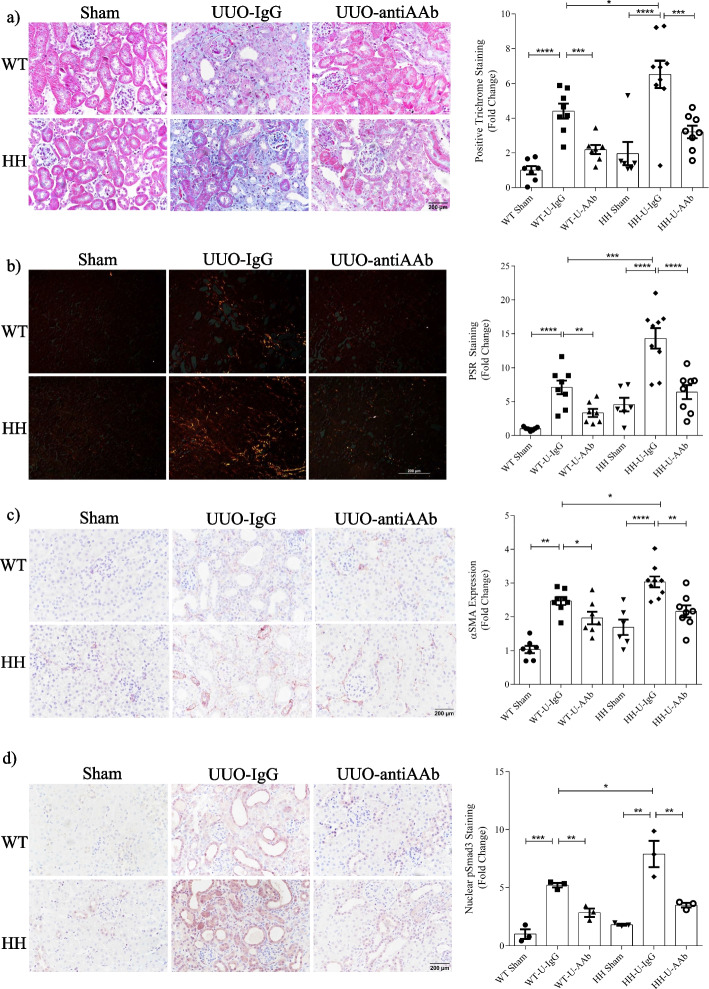
Augmented fibrosis in UUO by TGFβ1 overexpression is attenuated by actA neutralization. TGFβ1 overexpression worsened UUO-induced fibrosis as assessed by **a** Trichrome and **b** PSR. Both were attenuated by actA neutralization. **c** TGFβ1 overexpression augmented UUO-induced expression of the fibroblast marker αSMA, which was attenuated by actA inhibition. **d** Nuclear levels of phosphorylated Smad3 were also augmented by TGFβ1 overexpression after UUO and this was inhibited by actA neutralization (n = 6–9) *, **, ***, ****P < 0.05, 0.01, 0.001, 0.0001; one-way ANOVA with Tukey’s multiple comparisons post hoc tests

We next assessed effects of both TGFβ1 overexpression and actA neutralization on renal Smad3 activation, MRTF-A expression and fibrosis. Figure 6d shows the increased Smad3 activation and MRTF-A expression induced by UUO was augmented in H/H mice, as was fibronectin expression. These were attenuated by actA inhibition. Masson’s trichrome (Fig. 7a) and PSR (Fig. 7b) stained for collagen in blue and orange, respectively, showing development of significant interstitial fibrosis in WT mice which increased further with TGFβ1 overexpression. Fibrosis was significantly reduced by actA neutralization. Similar findings were observed for αSMA (Fig. 7c) and nuclear phosphorylated Smad3 (Fig. 7d). Importantly, actA neutralization in TGFβ1 overexpressing mice reduced all of these to levels seen in treated WT mice, supporting an important role for actA in mediating TGFβ1 profibrotic effects in vivo.

## Discussion

Regardless of etiology, CKD is characterized by excessive accumulation of extracellular matrix and thus fibrosis, with TGFβ1 known to be a major mediator [5]. While inhibition of TGFβ1 attenuates renal fibrosis and progressive loss of kidney function in animal models of CKD [5], adverse effects limit the clinical utility of its inhibition [6]. Activins are, however, emerging as potential important mediators of the TGFβ1 profibrotic effects and thereby may represent a more tolerable anti-fibrotic treatment alternative. The mechanisms through which activins sustain TGFβ1 profibrotic effects, however, have been unclear. Here, we show that actA is required for longer-term TGFβ1-induced fibrotic responses at least in part through regulation of both Smad3 and MRTF-A signaling.

Previous studies showed that follistatin inhibits TGFβ1 profibrotic signaling in numerous cell types, including renal fibroblasts and MC [17, 18]. Although follistatin inhibits several members of the TGFβ superfamily, including TGFβ3, BMPs 2, 4, 5, 7, 8 and GDF8, 11, it has greatest neutralizing potency against activins A and B, with highest potency against actA [7, 14]. Although future studies should also evaluate a potential role for other ligands neutralized with higher affinity by follistatin, namely GDF8 (myostatin), our initial studies focused on the assessment of these two activins. We find that TGFβ1 increases secretion primarily of actA, which itself induces Smad3 activation and stimulates production of profibrotic proteins. ActA secretion in response to TGFβ1 was also shown in other cell types [31, 32]. Importantly, specific actA neutralization recapitulated follistatin effects, while actB neutralization was ineffective. These studies identify actA as the primary activin mediator of TGFβ1 profibrotic effects.

The TGFβ3 isoform was also identified to be profibrotic in kidney cells [33]. Interestingly, much of this effect was mediated by TGFβ1. We thus assessed whether TGFβ3 could induce actA production as we found for TGFβ1. Additional file 1: Fig. S7 shows a small increase in actA synthesis and secretion in MC in response to TGFβ3. These data suggest that the contribution of TGFβ3 to increased actA levels would be much less significant than that of TGFβ1.

Although TGFβ1 and actA bind to distinct receptors, Smad3 mediates the profibrotic effect of both cytokines. The question thus arises as to why actA is required for TGFβ1 profibrotic effects. We first showed that the kinetics of activation are different: TGFβ1 is a potent early activator while activin effects are less potent, but more sustained. Interestingly, while early TGFβ1 activation of Smad3 (30 min) was unaffected by follistatin, in keeping with its inability to directly neutralize this ligand, follistatin attenuated later (24 h) Smad3 activation. The key role for actA was confirmed by its specific neutralization. It is known that cells become refractory to TGFβ1 after acute stimulation through receptor depletion after ligand binding, with relative unresponsiveness after 6–8 h of initial stimulation. Slow receptor replenishment ultimately restores signaling ability [23]. Thus, longer-term signaling by TGFβ1 is influenced by receptor dynamics. In keeping with this, we observed that cells became refractory to repeated TGFβ1 stimulation while remaining sensitive to actA, supporting the notion that actA may sustain TGFβ1-initiated Smad3 activation.

With longer-term exposure, TGFβ1 is known to increase TRII expression [34]. Interestingly, we found that this is attenuated by actA inhibition, with no effect on TRI. Induction of TRII cell surface expression, most relevant for ligand binding and cell signaling [35], was also decreased by actA inhibition. Increased cell surface TRII may also occur rapidly (within 5–30 min) via translocation from intracellular stores, not associated with altered total cellular levels [35]. In contrast, we found an increase in total cellular TRII with acute (30 min) TGFβ1 treatment that was abrogated by prior inhibition of actA, suggesting an important contribution by basal actA to the ability of MC to acutely respond to TGFβ1. Supporting this, inhibition of basal actA attenuated TGFβ1-induced Smad3 activation. Given that actA preincubation for 24 h did not alter cell sensitivity to TGFβ1, these data suggest that basal actA levels are sufficient to support TGFβ1 signaling. Interestingly, assessment of the effects of TGFβ1 on expression of actA type I and IIA and B receptors reveal that all were transcriptionally increased by 24 h of treatment (Additional file 1: Fig. S8). This was prevented by follistatin, suggesting that TGFβ1 maintains its profibrotic signaling through enhanced activin signaling.

While TGFβ1- and actA-induced Smad3 activation were not additive, actA augmented αSMA induction by TGFβ1, suggesting that actA also regulates noncanonical signaling. We examined MRTF-A given its well-established role in αSMA upregulation [26] and evidence that actA signals through MRTF-A in neuronal cells [36]. Our data showing that actA alone induces MRTF-A nuclear translocation and that actA inhibition prevents nuclear MRTF-A translocation by TGFβ1 confirm additional regulation of noncanonical TGFβ1 signaling by actA. Although the molecular mechanism is currently unknown, regulation of SCAI (suppressor of cancer cell invasion), a nuclear negative regulator of MRTF [26] may be relevant. Indeed, actA increased MRTF activity in neuronal cells by promoting SCAI nuclear export [36], and TGFβ1 attenuated SCAI expression in kidney tubular cells [37]. Further studies are needed to address this.

Although not explored in this manuscript, the potential contribution of actA regulation of other noncanonical signaling pathways should be considered. Indeed, data on the regulation of several non-canonical pathways by activin A are emerging, similarly to that seen with TGFβ1, with the specific pathway involved depending on cell type and stimulus. In addition to activation of MRTF-A, activin A was also shown in cardiac fibroblasts and a mouse fibroblast cell line to regulate cell proliferation and differentiation through the mitogen activated protein kinases p38 and extracellular signal-regulated kinases 1/2 (ERK1/2) [38, 39]. In ovarian cancer cells, the activin A migratory effect was dependent on Akt, Erk and Rac1 activation [40]. In endometrial mesenchymal stem cells, on the other hand, STAT3 activation by activin A, but not Akt, p38 or JNK, mediated profibrotic CTGF expression [22]. Future studies would explore the relevance of these and other noncanonical signaling mediators in the regulation of TGFβ1 profibrotic effects by actA.

TGFβ1 also leads to the activation of Smad2. In kidney tubular epithelial cells, Smad2 was found to protect against TGFβ1-mediated fibrosis by counteracting TGFβ1/Smad3 signaling, and tubular Smad2 deletion exacerbated fibrosis in the UUO model [41]. We assessed the effects of follistatin and actA on Smad2 phosphorylation by TGFβ1 after 24 h. Additional file 1: Fig. S9 shows that unlike the inhibition of Smad3 we had observed, Smad2 remained unaffected. This is of interest, suggesting that actA discriminates towards potentiating profibrotic Smad3 activation. Thus, actA inhibition would attenuate Smad3 activation while enabling ongoing antifibrotic Smad2 activity.

Additional mechanisms by which actA may regulate TGFβ1 signaling should also be considered. For example, actA decreased Smad7 expression in granulosa cells [42]. Suppression of this inhibitory Smad may enable and/or extend Smad3 activation by TGFβ1. Indeed, its deletion enhanced fibrosis after UUO [43]. Studies in zebrafish also show that cells respond to TGFβ superfamily ligands according to the total cumulative ligand dose and duration of exposure [44]. Increased actA may thus provide an overall stronger signal to augment TGFβ1 responses. Additionally, unlike TGFβ1, actA possesses long-range signaling ability via diffusion in tissue [45]. This may help to explain why fibroblast deletion of TRII did not attenuate fibrosis in mouse models of CKD [46]. Here, actA originating from non-adjacent cell types such as tubular cells may signal to fibroblasts [47]. Indeed, in cultured proximal tubular cells, TGFβ1 increased actA (but not B) production (Additional file 1: Fig. S6). Finally, as organs fibrose, their stiffness increases [48]. Interestingly, increased tumor stiffness promoted actA secretion in response to TGFβ1 [49], suggesting that as fibrosis progresses and organ stiffness increases, actA secretion may be augmented to perpetuate the maladaptive profibrotic response. How actA transcriptional regulation by TGFβ1 occurs is not well documented and requires further investigation.

Divergent signaling downstream of TGFβ1 has also been observed in some settings, in which actA was shown to mediate only specific TGFβ1 effects while others were independent of this cytokine. For example, in colon cancer cells, growth suppression by TGFβ1 was dependent on actA, while cellular migration was not. Furthermore, levels of both ligands together were more predictive of outcomes in patients with colon cancer compared with individual cytokine levels, suggesting an additive effect on pathology [31]. ActA and TGFβ1 may also have independent and opposite effects, such as on the expression of p21 in these cancer cells. Here, actA suppressed p21 expression through PI3K/Akt activation, while TGFβ1 enhanced it through Erk and Smad4 signaling [50]. The requirement for actA in mediating TGFβ1 profibrotic effects thus is not recapitulated for all of TGFβ1 downstream outcomes, and may differ between cell types.

To date, no in vivo studies have established the requirement for actA in TGFβ1-induced fibrosis. To address this question, we used mice engineered to overexpress the TGFβ1 transcript [21]. While previous studies showed that local renal TGFβ1 overexpression results in fibrosis [51, 52], the degree of overexpression in our model was insufficient to cause baseline histologic changes [21]. Similarly, neither circulating nor renal actA levels were increased. However, the importance of actA as a mediator of TGFβ1-induced renal fibrosis was apparent after induction of renal injury with UUO. In this model, increased TGFβ1 expression is well established, as is the elevation in both circulating and renal actA levels [30, 53]. Our data confirmed this, showing a further increase in renal actA with TGFβ1 overexpression which was associated with worsened pathology after UUO. A previous study has shown inhibition of collagen accumulation and fibrosis by follistatin in this model [54]. Our data here importantly emphasize the major contribution of actA to this observation given that actA neutralization attenuated Smad3 activation and fibrosis to levels seen in WT mice. With a significant reduction in αSMA, actA effects on fibroblasts (Additional file 1: Figs. S2, S5) were likely a major contributor to the phenotype.

**Fig. 8:**
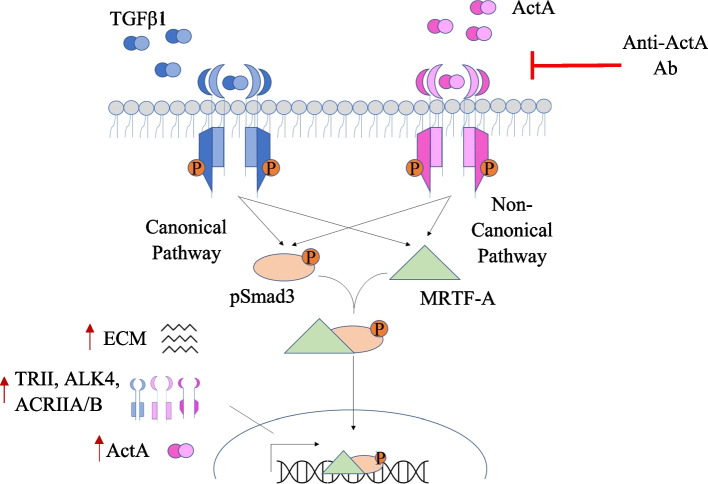
A schematic representation showing ActA-stimulated Smad3 and MRTF-A signaling converge to enable sustained TGFβ1 profibrotic responses. ActA regulation of TRII and actA receptors, as well as ongoing actA receptor responsiveness to its ligand in contrast to the refractoriness of TGFβ1 receptors to TGFβ1, are important in maintaining profibrotic TGFβ1 signaling. Neutralization of actA attenuates TGFβ1-induced fibrosis

In summary (Fig. 8), we show that actA regulates TGFβ1 canonical (Smad3) and noncanonical (MRTF-A) signaling to enable its longer-term fibrotic effects both in vitro and in vivo. Altogether, actA inhibition offers a promising antifibrotic target that may be more clinically tolerated than TGFβ1 inhibition and may avoid some of the adverse effects seen with alternate strategies that more broadly inhibit numerous TGFβ1 family ligands [10, 55, 56]. Notably, human neutralizing actA antibodies have already been developed to treat the disorder fibrodysplasia ossificans progressiva, characterized by excessive actA activity [10, 57], thus supporting clinical translatability of this approach.

## Supplementary Information

**Additional file 1: Figure S1.** Follistatin does not inhibit early Smad3 activation by TGFβ1. MC were treated with follistatin for 30 min prior to the addition of TGFβ1 for 30 min. Smad3 phosphorylation was assessed by immunoblotting. ****P < 0.0001; one-way ANOVA with Tukey’s multiple comparisons post hoc test. **Figure S2.** Activin A supports TGFβ1 profibrotic effects in renal fibroblasts. Follistatin (FST) and actA neutralization attenuated TGFβ1-induced fibronectin (FN), α-smooth muscle actin (αSMA) and connective tissue growth factor (CTGF) upregulation as well as Smad3 activation at 48 h (n = 5–8). *, **, ***, ****P < 0.05, 0.01, 0.001, 0.000; one-way ANOVA with Tukey’s multiple comparisons post hoc test. **Figure S3.** Confirmation of specificity of the actA neutralizing antibody in MC. The neutralizing antibody for actA prevents actA, but not actB, induction of Smad3 transcriptional activity at 24 h as assessed by the CAGA12 luciferase reporter (n = 6). **, ****P < 0.01, 0.0001; one-way ANOVA with Tukey’s multiple comparisons post hoc test. **Figure S4.** ActA inhibition prevents profibrotic gene upregulation by TGFβ1 in MC. Increased fibronectin (FN), collagen Iα1 (ColI) and α-smooth muscle actin (αSMA) transcripts by 24 h of TGFβ1 were attenuated with a neutralizing actA antibody (n = 6). **, ***P < 0.01, 0.001; one-way ANOVA with Tukey’s multiple comparisons post hoc test. **Figure S5.** ActA enables renal fibroblast proliferation, migration and gel contraction. (a) Increased cell proliferation induced by TGFβ1 (24 h) is significantly decreased by both follistatin and a neutralizing actA antibody (n = 3). (b) TGFβ1 increased wound closure after 24 h in a scratch assay. This was inhibited by both follistatin and an actA neutralizing antibody (n = 3). (c) TGFβ1 induced collagen gel contraction after 72 h, which was prevented by follistatin and an actA neutralizing antibody (n = 3). *, **P < 0.05, 0.01; one-way ANOVA with Tukey’s multiple comparisons post hoc test. **Figure S6.** TGFβ1 induces activin A, but not activin B production in human proximal tubular cells. TGFβ1: (a) upregulates actA, but not actB, transcript levels at 24 h (n = 6–9); (b) induces actA secretion in a dose-dependant manner (0.5–5 ng/ml TGFβ1) (n = 3); (c) has no effect on actB secretion at 24 h (n = 3). **, ***, ****P < 0.01, 0.001, 0.0001; one-way ANOVA with Tukey’s multiple comparisons post hoc test. **Figure S7.** TGFβ3 induces actA synthesis and secretion in MC. ELISA shows a small increase in both a) actA secretion into the medium (n = 3) and b) cellular actA levels in response to 24 h of TGFβ3 treatment (n = 3). **P < 0.01; one-way ANOVA with Tukey’s multiple comparisons post hoc test. **Figure S8.** In MC, 24 h TGFβ1 increases transcript levels for activin type I and II receptors ALK4 and ActRIIA/B respectively. This is dependent on activin signaling itself as it is inhibited by follistatin (n = 3). *, **, ***P < 0.05, 0.01, 0.001; one-way ANOVA with Tukey’s multiple comparisons post hoc test. **Figure S9.** ActA does not regulate the activation of Smad2 by TGFβ1 in MC. Smad2 activation was assessed by its phosphorylation after 24 h of TGFβ1. Neither follistatin nor the actA neutralizing antibody prevented its activation (n = 3); one-way ANOVA with Tukey’s multiple comparisons post hoc test.

## Data Availability

All data are included in the manuscript and supporting information.

## Abbreviations

ActA: Activin A
ActB: Activin B
ALK4: Activin-like kinase 4
ALK7: Activin-like kinase 7
ActRIIA: Activin receptor type-IIA
ActRIIB: Activin receptor type-IIB
BMP: Basic metabolic panel
CKD: Chronic kidney disease
CTGF: Connective tissue growth factor
p21: Cyclin-dependent kinase inhibitor 1
FN: Fibronectin
FST: Follistatin
GDF: Growth and differentiation factor
H/H: TGFβ1 overexpression
MRTF-A: Myocardin related transcription factor A
pSmad3: Phosphorylated Suppressor of Mothers against Decapentaplegic
PI3K: Phosphatidylinositol-3-kinase
SCAI: Suppressor of cancer cell invasion protein
Smad2/3: Suppressor of Mothers against Decapentaplegic 2/3
TRI: TGFβ-receptor type I
TRII: TGFβ-receptor type II
UUO: Unilateral ureteral obstruction
TGFβ: Transforming growth factor beta
