# Supplementary figures and images for: A therapeutic target for CKD: activin A facilitates TGFβ1 profibrotic signaling
Source: Cell Mol Biol Lett. 2023 Jan 30;28:10. doi: 10.1186/s11658-023-00424-1 (PMC9885651; doi:10.1186/s11658-023-00424-1)

## Figure S1

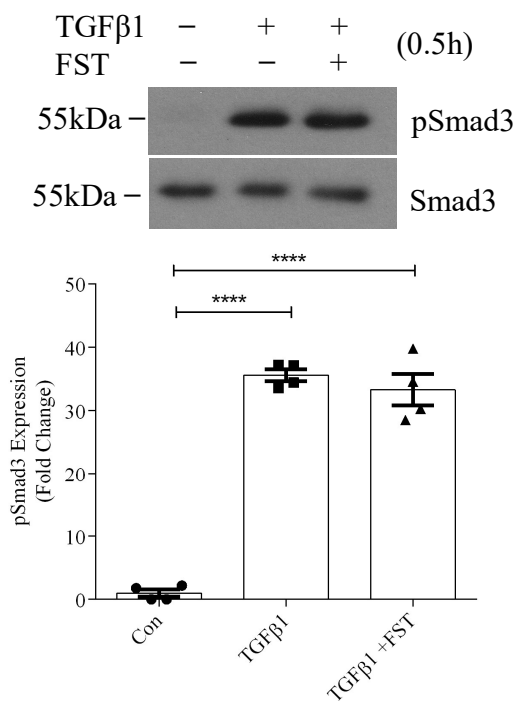

### Figure S3

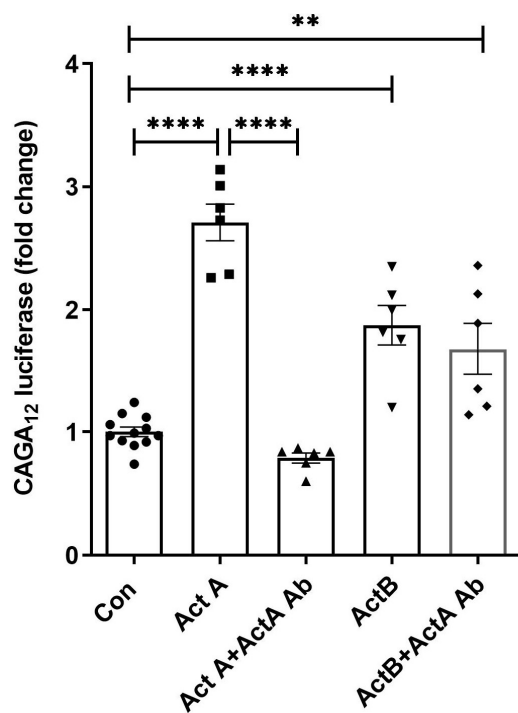

### Figure S2

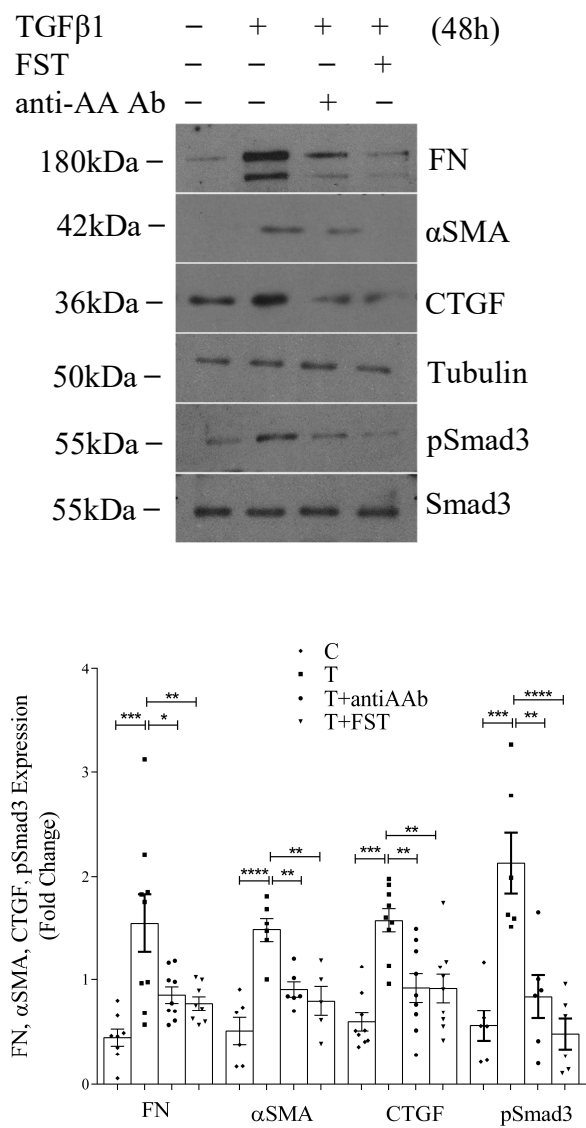

Figure S4

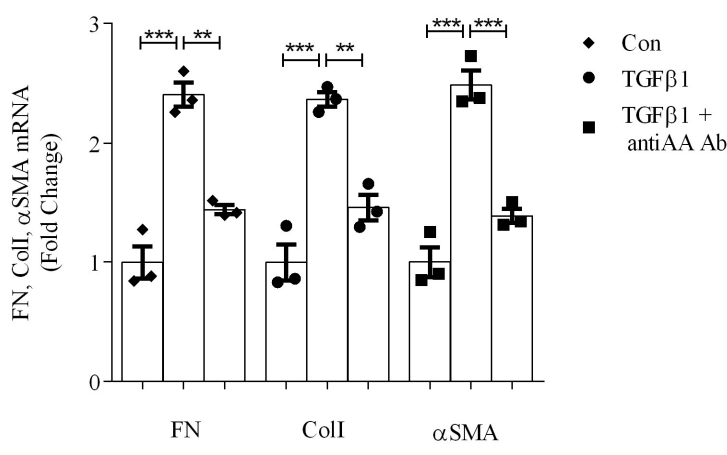

Figure S5

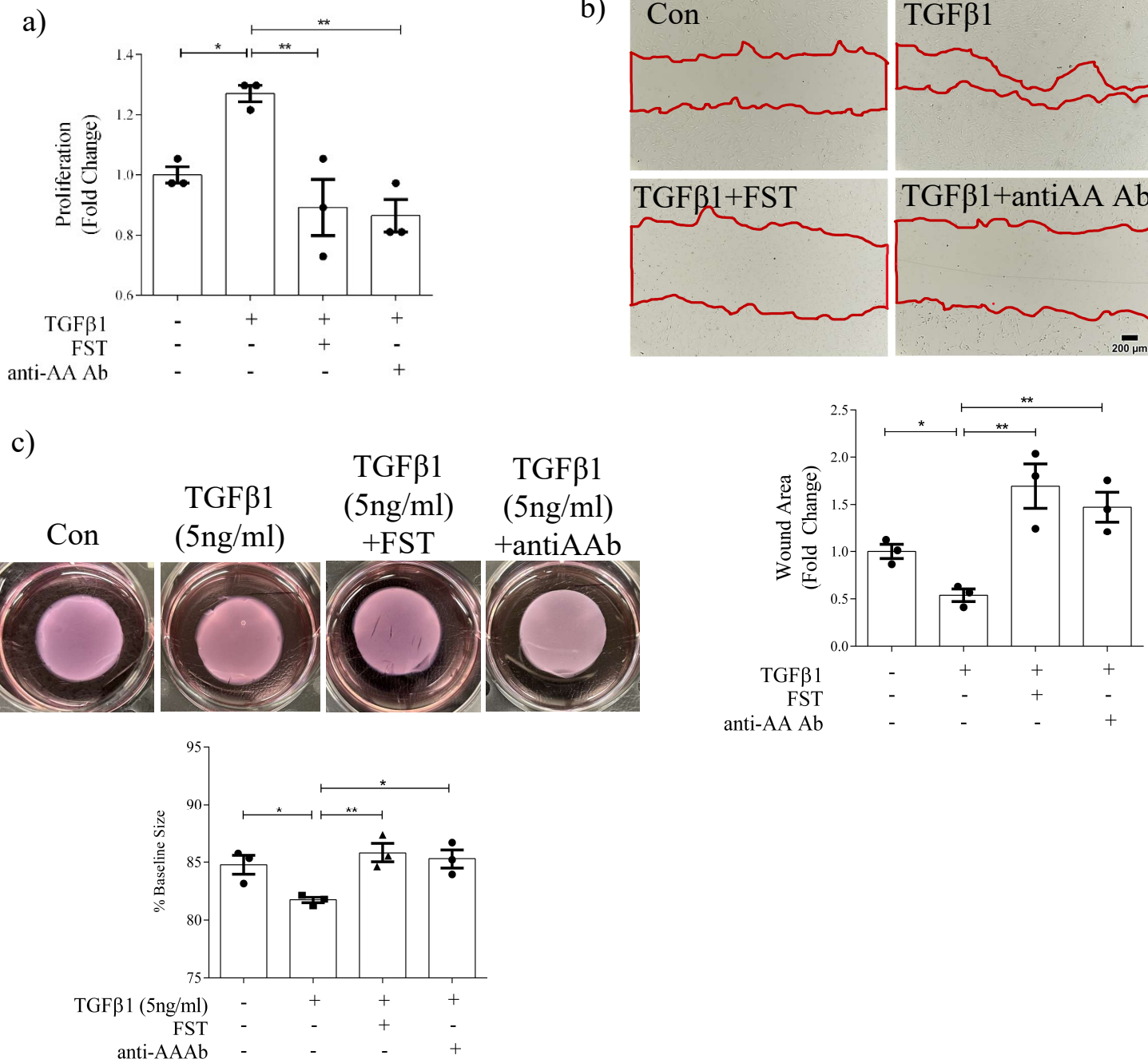

**Figure S6**

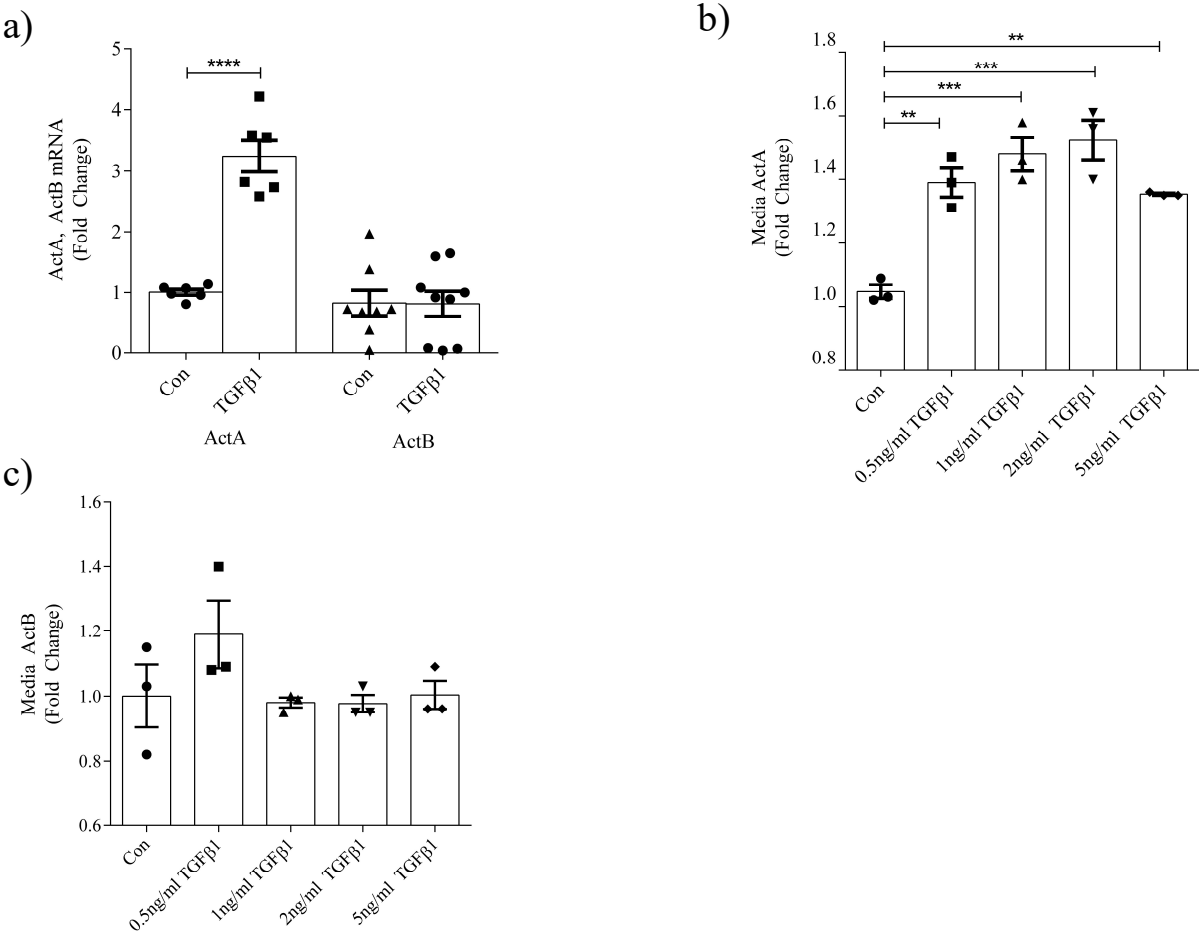

**Figure S7**

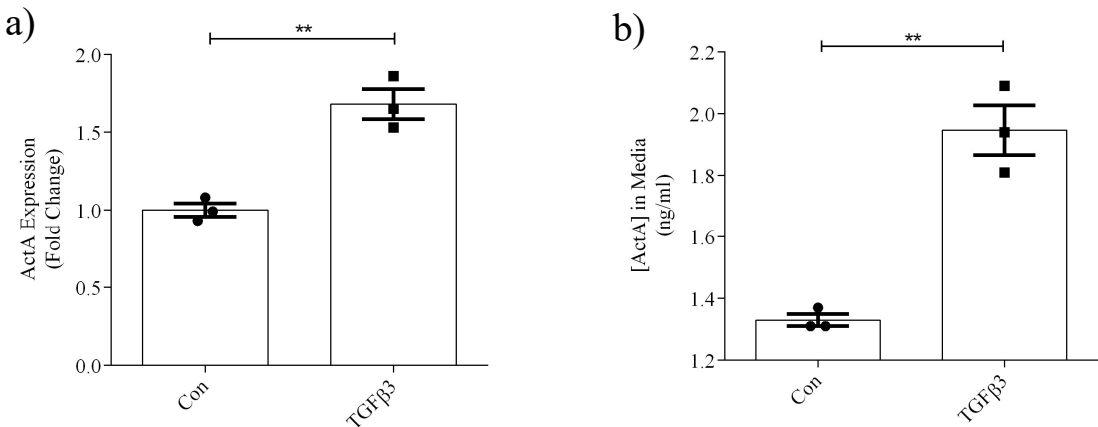

Figure S8

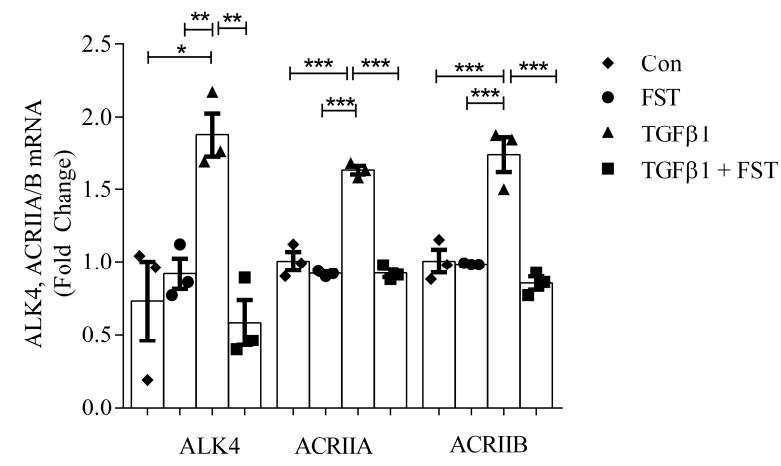

Figure S9

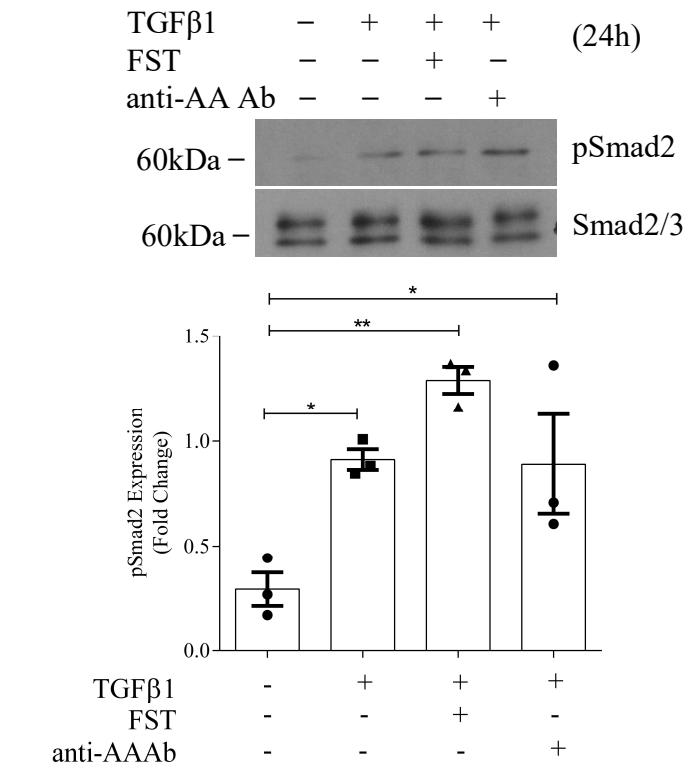

Supplement: Supplementary file 1 — Additional file 1: Figure S1. Follistatin does not inhibit early Smad3 activation by TGFβ1. MC were treated with follistatin for 30 min prior to the addition of TGFβ1 for 30 min. Smad3 phosphorylation was assessed by immunoblotting. ****P < 0.0001; one-way ANOVA with Tukey’s multiple comparisons post hoc test. Figure S2. Activin A supports TGFβ1 profibrotic effects in renal fibroblasts. Follistatin (FST) and actA neutralization attenuated TGFβ1-induced fibronectin (FN), α-smooth muscle actin (αSMA) and connective tissue growth factor (CTGF) upregulation as well as Smad3 activation at 48 h (n = 5–8). *, **, ***, ****P < 0.05, 0.01, 0.001, 0.000; one-way ANOVA with Tukey’s multiple comparisons post hoc test. Figure S3. Confirmation of specificity of the actA neutralizing antibody in MC. The neutralizing antibody for actA prevents actA, but not actB, induction of Smad3 transcriptional activity at 24 h as assessed by the CAGA12 luciferase reporter (n = 6). **, ****P < 0.01, 0.0001; one-way ANOVA with Tukey’s multiple comparisons post hoc test. Figure S4. ActA inhibition prevents profibrotic gene upregulation by TGFβ1 in MC. Increased fibronectin (FN), collagen Iα1 (ColI) and α-smooth muscle actin (αSMA) transcripts by 24 h of TGFβ1 were attenuated with a neutralizing actA antibody (n = 6). **, ***P < 0.01, 0.001; one-way ANOVA with Tukey’s multiple comparisons post hoc test. Figure S5. ActA enables renal fibroblast proliferation, migration and gel contraction. (a) Increased cell proliferation induced by TGFβ1 (24 h) is significantly decreased by both follistatin and a neutralizing actA antibody (n = 3). (b) TGFβ1 increased wound closure after 24 h in a scratch assay. This was inhibited by both follistatin and an actA neutralizing antibody (n = 3). (c) TGFβ1 induced collagen gel contraction after 72 h, which was prevented by follistatin and an actA neutralizing antibody (n = 3). *, **P < 0.05, 0.01; one-way ANOVA with Tukey’s multiple comparisons post hoc test. Figure S6 [file 11658_2023_424_MOESM1_ESM.pdf]
